# Characterizing the fecal bacteria and archaea community of heifers and lactating cows through 16S rRNA next-generation sequencing

**DOI:** 10.1007/s13353-020-00575-3

**Published:** 2020-08-26

**Authors:** Filippo Cendron, Giovanni Niero, Gabriele Carlino, Mauro Penasa, Martino Cassandro

**Affiliations:** Department of Agronomy, Food, Natural Resources, Animals and Environment, University of Padova, Viale dell’Università, 16, 35020 Legnaro, PD Italy

**Keywords:** Dairy and dual-purpose breeds, Efficiency, Feces, Microbiome

## Abstract

The aim of this study was to describe the fecal bacteria and archaea composition of Holstein-Friesian and Simmental heifers and lactating cows, using 16S rRNA gene sequencing. Bacteria and archaea communities were characterized and compared between heifers and cows of the same breed. Two breeds from different farms were considered, just to speculate about the conservation of the microbiome differences between cows and heifers that undergo different management conditions. The two breeds were from two different herds. *Firmicutes*, *Bacteroidetes*, *Actinobacteria*, and *Proteobacteria* were the most abundant phyla in all experimental groups. Alpha- and beta-diversity metrics showed significant differences between heifers and cows within the same breed, supported by principal coordinate analysis. The analysis of Holstein-Friesian fecal microbiome composition revealed 3 different bacteria families, 2 genera, and 2 species that differed between heifers and cows; on the other hand, Simmental heifers and cows differed only for one bacteria family, one archaeal genus, and one bacteria species. Results of the present study suggest that fecal communities of heifers and cows are different, and that fecal microbiome is maintained across experimental groups.

## Introduction

The fecal microbiome consists of a complex community of microorganisms and represents a central issue in relation to cattle welfare and feed efficiency. In particular, the associations between fecal microbiome and animal health have been shown in the intestinal microbiota of calves (Oikonomou et al. 2013). The main factor that influences fecal microbiome composition is animal diet. Callaway et al. (2010) carried out an evaluation of bacterial diversity of 6 cattle (3 Jersey cows and 3 Angus steers) through a comparison of 3 different diets in terms of amount of dried distillers grain; Shanks et al. (2011) analyzed the structure of fecal community in 30 adult beef cattle equally divided in 3 diet groups; and Rice et al. (2012) evaluated the influence of different types and amount of distillers grains on fecal microbial assemblages in 20 crossbreed cattle. The forage to concentrates ratio in the diet is the major factor affecting fecal microbiome composition in cattle (Kim et al. 2014). According to the meta-analysis of Kim and Wells (2016), the fecal cattle microbiome is composed of 10 phyla, 17 classes, 28 orders, 59 families, and 100 genera. *Firmicutes* is the most represented phylum, followed by *Bacteroidetes* and *Proteobacteria*. Within *Firmicutes*, *Clostridia* and *Fecalibacterium* are the largest class and genus, respectively. Within *Bacteroidetes*, *Bacteroidia* is the largest class and *Prevotella* the largest genus. Finally, *Proteobacteria* includes *Gammaproteobacteria* and *Succinivibrio* as the most abundant class and genus, respectively. To investigate the microbiome in cattle, most studies have used DNA-based methodologies such as Sanger sequencing technology, quantitative real-time polymerase chain reaction, and phylogenetic microarrays (Kim et al. 2017; Mende et al. 2012). Currently, the next-generation sequencing (NGS) is considered the most reliable approach to evaluate the diversity of bacteria, both in rumen and feces of cattle (Kim et al. 2017). The 16S rRNA is widely used as reference gene to determine the composition of bacterial community due to its phylogenetic variability (Tringe and Hugenholtz 2008); indeed, it includes 9 hypervariable regions and 10 conserved regions. The conserved regions (C1 to C10) are shared among bacterial and archaeal species, whereas 16S rRNA hypervariable regions (V1 to V9) are different. The latter can be targeted to identify individual bacterial or archaeal species using PCR with species-specific primers for the 16S rRNA gene. Data analysis assigns 16S rRNA sequences to operational taxonomic units (OTUs) that can be identified according to available database. The literature reports differences of fecal microbiome composition within beef cattle breeds, across dairy and beef cattle breeds, and within crossbreed cattle (Durso et al. 2012). Comparisons between heifers and cows of dairy and dual-purpose cattle breeds are currently lacking. The aim of this study was to characterize and analyze the difference of the fecal microbiome community of heifers and cows of dairy and dual-purpose cattle breeds, targeting the hypervariable regions of the bacterial 16S; moreover, we evaluated if the microbiome composition is conserved between the breeds that underwent to different management and diet composition. Gaining knowledge on these aspects is expected to be beneficial to investigate changes in methane emissions and variation of feed efficiency, as well as to develop non-invasive routine controls to evaluate animal welfare and health.

## Materials and methods

### Sample collection and DNA extraction

Fecal samples were collected through rectal picking during routine health monitoring of animals by authorized veterinarians of the Breeders Association of Veneto Region (Italy). Twenty individual samples (one sample per animal) from 2 single-breed herds (one rearing Holstein-Friesian and the other Simmental breed) were collected for microbiome analysis, considering two categories: cows and heifers. Animals were divided in 2 experimental groups: (1) Holstein-Friesian heifers (HFH, *n* = 5) and Holstein-Friesian cows (HFC, *n* = 5); (2) Simmental heifers (SIH, *n* = 5) and Simmental cows (SIC, *n* = 5). The sample size was chosen after a literature review (Callaway et al. 2010; Sandri et al. 2018). A description of rations used in the 2 farms is presented in Table 1.

**Table 1:** Diet composition (kg/day) in each farm and cattle category

	Farm 1		Farm 2	
	HFC	HFH	SIC	SIH
Corn silage	22	8	25	7.5
Wheat silage	-	3.5	-	-
Corn mash	6	-	-	-
Alfalfa hay	4.5	-	5	-
Grass hay	1.5	3.5	2	2
Soybean meal	3.3	1	-	-
Soybean flakes	0.6	-	-	-
Corn grain	2	-	-	-
Straw wheat	0.5	-	-	5.3
Corn meal	-	-	1	-
Protein supplement^a^	-	-	4	2.5
Mineral-vitamin premix	0.5	0.25	0.5	-
Energetic supplement^b^	-	-	3.8	-
Hydrogenated fat	0.3	-	0.3	-
Extruded flaxseed	0.25	-	-	-
Sodium bicarbonate	0.15	-	-	-
Yeast	-	-	0.05	-
Total mixed ration	41.6	16.25	41.65	17.3

*HFC*, Holstein-Friesian cows; *HFH*, Holstein-Friesian heifers; *SIC*, Simmental cows; *SIH*, Simmental heifers

^a^Sunflower seed flour, roasted soybean seeds, dehulled soy flour–based feed, maize, sugar cane molasses

^b^Maize, barley, sugar cane molasses

Feces were stored at −80 °C within 1 h from sampling. DNA extraction was performed through AllPrep PowerFecal® DNA/RNA Kit (Qiagen, Hilden, Germany), and the quantity and quality of total DNA were checked through spectrophotometer assay (Multiscan Sky, Thermo Fisher Scientific, USA).

### Next-generation sequencing

Total genomic DNA was amplified by using a standard protocol and modified primers (Takahashi et al. 2014). Amplicons were purified through magnetic beads Agencourt XP 0.8× (Beckman Coulter, Brea, CA, USA) and amplified through HiSeq by using Index Nextera XT kit (Illumina, San Diego, CA, USA). All amplified sequences were normalized by SequalPrep (Thermo Fisher, Waltham, MA, USA) and precipitated through magnetic beads Agencourt XP 0.8×. Libraries were loaded onto MiSeq (Illumina, San Diego, CA, USA) and sequenced following V3-300PE strategy.

### Statistical analyses

The OTUs obtained from 16S rRNA sequencing results were filtered for 0.005% frequency, and organized in an OTU table. The taxonomic survey was obtained from a cross comparison between the QIIME2 software package (http://qiime.org/) and the two databases SILVA v.1.132 and Geengenes v.13.8 (the last as a comparison); clustered OTUs were matched against references from databases. Alpha-diversity analyses were conducted considering Observed OTUs, Shannon Index, Pielou’s Evenness, and Faith’s Phylogenetic Diversity Index using QIIME2 platform. All sequences were clustered with representative OTUs and cleaned considering 97% of identity as cutoff. The statistical significance of each index was analyzed by Kruskal-Wallis non-parametric test, comparing cows and heifers within the same breed. Beta-diversity was calculated through the Bray-Curtis Metric, Jaccard Metric, and the UniFrac Metric (weighted and unweighted) to evaluate the dissimilarity and distance between the animals of the same breed. Dissimilarities in fecal bacteria and archaea communities were visualized using principal coordinate analysis (PCoA) method. The permutational analysis of variance (PERMANOVA) (Anderson 2005) and analysis of similarities (ANOSIM) were performed in R-vegan package adonis (Oksanen et al. 2017). Finally, differential abundance test was performed using ANCOM packages of R software (Team R, Core Team 2015). Significance was determined through *W* statistic, which indicates the number of times the null hypothesis was rejected. Positive values of *W* statistic correspond to more abundant taxa in the comparison of HFH vs HFC, and SIH vs SIC. The test was performed accounting for the percentile abundance.

## Results

### Taxonomic identification

The total OTUs obtained (2,302) were clustered trough SILVA and Geengenes for the taxonomic analysis; this identified the presence of 2 kingdoms, 14 phyla, 22 classes, 34 orders, 74 families, 212 genera, and 350 species, while the remaining sequences were not assigned to known phyla (Table 2).

**Table 2:** Average number of sequences (standard deviation) per taxon obtained from fecal samples of Holstein-Friesian heifers (HFH), Holstein-Friesian cows (HFC), Simmental heifers (SIH), and Simmental cows (SIC)

	HFH	HFC	SIH	SIC
Archaea	819 (371)	2,289 (731)	1,429 (503)	1,557 (539)
*Euryarchaeota*	819 (371)	2,289 (731)	1,429 (503)	1,557 (539)
*Methanobrevibacter*	700 (277)	2,132 (663)	1,200 (414)	1,389 (532)
*Methanocorpusculum*	73 (66)	17 (18)	77 (38)	11 (10)
*Methanosphaera*	22 (12)	141 (64)	74 (33)	157 (34)
Unclassified Methanobacteriaceae	13 (9)	-	73 (25)	-
Uncultured Methanomethylophilaceae	13 (7)	-	6 (5)	-
Bacteria	27,662	26,443	26,401	27,262
*Actinobacteria*	35 (16)	1,265 (589)	73 (41)	594 (399)
*Aeriscardovia*	-	14 (3)	-	3 (4)
*Arcanobacterium*	6 (7)	-	1(2)	-
*Atopobium*	3 (4)	5 (1)	3 (4)	9 (8)
*Bifidobacterium*	-	1,130 (565)	-	28 (26)
*Olsenella*	-	66 (42)	25 (34)	493 (357)
*Raoultibacter*	2 (3)	-	7 (5)	-
*Eggerthellaceae DNF00809*	10 (11)	13 (6)	22 (14)	13 (10)
Uncultured Eggerthellaceae	1 (2)	5 (6)	2 (3)	13 (8)
Unclassified Actinobacteria	12 (7)	31 (16)	14 (20)	35 (20)
*Bacteroidetes*	12,529 (610)	10,308 (753)	9,997 (586)	10,019 (581)
*Alistipes*	1,489 (181)	866 (207)	1,108 (248)	709 (230)
*Alloprevotella*	202 (72)	198 (146)	135 (86)	780 (348)
*Bacteroides*	1,715 (67)	2,807 (151)	1,759 (263)	2,350 (230)
*Odoribacter*	32 (8)	16 (24)	20 (11)	35 (16)
*Sanguibacteroides*	2 (4)	-	7 (6)	-
*Paludibacter*	-	15 (22)	-	-
*Parabacteroides*	11 (6)	37 (7)	19 (10)	103 (23)
*Prevotella*	137 (39)	479 (113)	78 (29)	193 (123)
*Prevotellaceae UCG-001*	82 (32)	258 (50)	74 (30)	150 (58)
*Prevotellaceae UCG-003*	988 (76)	1,908 (100)	704 (160)	1,172 (243)
*Prevotellaceae UCG-004*	1,168 (151)	183 (147)	1,308 (264)	532 (81)
*Prevotellaceae Ga6A1 group*	6 (7)	25 (21)	18 (14)	33 (27)
*Rikenellaceae RC9 gut group*	2,380 (158)	2,015 (254)	2,238 (313)	1,918 (229)
*Rikenellace dgA-11 gut group*	952 (183)	70 (83)	622 (88)	132 (79)
Uncultured Bacteroidales F082	558 (120)	8 (12)	773 (117)	87 (38)
Uncultured Bacteroidales gir-aah93h0	335 (116)	235 (233)	177 (32)	335 (240)
Uncultured Bacteroidales M2PB4-65 termite group	169 (183)	-	234 (192)	-
Uncultured Bacteroidales p-251-o5	118 (65)	21 (41)	169 (81)	125 (123)
Uncultured Bacteroidales p-2534-18B5 gut group	398 (185)	-	-	235 (216)
Uncultured Bacteroidales RF16 group Paludibacter sp.	36 (20)	-	30 (28)	19 (6)
Uncultured Bacteroidales RF16 group Porphyromonadaceae bacterium	225 (38)	196 (102)	259 (179)	218 (167)
Uncultured Barnesiellaceae	29 (11)	46 (23)	21 (7)	61 (44)
Uncultured Bacteroidales RF16 group Uncultured bacterium	43 (16)	22 (22)	165 (163)	35 (23)
Uncultured Bacteroidales UCG-001	49 (10)	-	76 (17)	25 (9)
Uncultured Bacteroidales	-	-	31 (8)	11 (14)
Uncultured Dysgonomonadaceae	149 (65)	82 (95)	316 (208)	198 (129)
Uncultured Flavobacteriaceae	702 (130)	394 (77)	163 (53)	332 (106)
Uncultured Muribaculaceae	213 (139)	60 (34)	31 (9)	17 (11)
Unclassified Bacteroidetes	341 (91)	367 (326)	166 (93)	195 (106)
*Chloroflexi*	3 (4)		34 (12)	
*Flexilinea*	3 (4)	-	34 (12)	-
*Cyanobacteria*	396 (174)	147 (212)	439 (146)	140 (84)
Uncultured Cyanobacteria	371 (162)	147 (212)	423 (162)	140 (84)
Unclassified Cyanobacteria	24 (25)	-	16 (18)	-
*Elusimicrobia*	32 (20)	3 (6)	39 (31)	2 (4)
*Eluisimicrobium*	32 (20)	3 (6)	39 (31)	2 (4)
*Epsilonbacteraeota*	7 (16)	1 (1)	2 (4)	1 (3)
*Campylobacter*	7 (16)	1 (1)	2 (4)	1 (3)
*Fibrobacteres*	156 (51)	-	511 (149)	71 (70)
*Fibrobacter*	156 (51)	-	511 (149)	71 (70)
*Firmicutes*	13,285 (1130)	13,568 (546)	13,216 (869)	15,380 (1022)
*Acetitomaculum*	17 (14)	47 (21)	65 (12)	65 (10)
*Aerococcus*	6 (7)	3 (8)	-	-
*Agathobacter*	10 (14)	170 (78)	-	77 (70)
*Anaerorhabdus furcosa group*	28 (14)	25 (13)	17 (10)	20 (7)
*Anaerosporobacter*	51 (21)	18 (17)	71 (98)	20 (31)
*Anaerostipes*	-	22 (27)	-	-
*Anaerovibrio*	-	97 (107)	-	29 (30)
*Anaerovorax*	30 (11)	16 (14)	42 (8)	200 (52)
*Angelakisella*	19 (4)	-	8 (3)	-
*Blautia*	7 (10)	137 (59)	24 (14)	104 (37)
*Breznakia*	1 (2)	21 (16)	1 (1)	3 (3)
*Butyricicoccus*	5 (12)	7 (4)	-	6 (4)
*Butyrivibrio*	8 (8)	19 (12)	2 (3)	12 (12)
*Candidatus Soleaferrea*	107 (20)	42 (14)	138 (29)	90 (10)
*Candidatus Stoquefichus*	-	149 (121)	-	-
*Caproiciproducens*	7 (9)	1 (1)	11 (10)	8 (8)
*Cellulosilyticum*	52 (69)	31 (23)	5 (4)	64 (33)
*Christensenellaceae R-7 group*	498 (134)	765 (213)	797 (156)	905 (403)
*Clostridium*	173 (11)	307 (271)	41 (14)	482 (262)
*Coprobacillus*	1 (3)	42 (9)	8 (3)	6 (8)
*Coprococcus*	17 (14)	121 (53)	37 (40)	122 (61)
*Defluvitaleaceae UCG-011*	22 (9)	14 (8)	8 (9)	13(11)
*Dielma*	7 (5)	13 (8)	12 (7)	15 (3)
*Dorea*	56 (30)	90 (34)	71 (23)	78 (39)
*Eisenbergiella*	-	4 (5)	1 (1)	2 (5)
*Erysipelatoclostridium*	1 (3)	42 (9)	8 (3)	42 (12)
*Erysipelotrichaceae UCG-002*	-	12 (16)	-	-
*Erysipelotrichaceae UCG-004*	10 (14)	104 (41)	-	24 (30)
*Erysipelotrichaceae UCG-006*	-	-	-	16 (17)
*Erysipelotrichaceae UCG-007*	-	-	-	27 (18)
*Erysipelotrichaceae UCG-009*	-	4 (3)	-	-
*Eubacterium brachy group*	56 (31)	66 (10)	72 (8)	80 (21)
*Eubacterium halii group*	7 (7)	1 (1)	22 (5)	37 (33)
*Eubacterium nodatum group*	42 (35)	31 (10)	80 (22)	71 (43)
*Eubacteriu ruminantium group*	2 (4)	23 (22)	11 (11)	32 (22)
*Eubacterium ventriosum group*	2 (2)	-	11 (8)	-
*Fecalibacterium*	-	5 (2)	-	4 (2)
*Fecalitalea*	9 (8)	65 (51)	2 (3)	7 (7)
*Family XIII AD3011*	273 (50)	140 (18)	273 (26)	200 (52)
*Family XIII UCG-001*	3(4)	6 (2)	3 (4)	10 (2)
*Flavonifractor*	14 (32)	66 (36)	30 (19)	57 (21)
*Fourneriella*	-	13 (10)	-	12 (3)
*Howardella*	4 (6)	20 (8)	18 (10)	45 (33)
*Intestinimonas*	3 (7)	-	6 (6)	-
*Lachnoclostridium*	51 (35)	24 (29)	55 (16)	44 (24)
*Lachnospira*	8 (19)	-	-	-
*Lachnospiraceae AC2044 group*	48 (20)	274 (118)	44 (18)	68 (70)
*Lachnospiraceae FCS020 group*	5 (5)	5 (7)	7 (8)	-
*Lachnospiraceae FE2018 group*	-	4 (3)	2 (3)	2 (2)
*Lachnospiraceae NK3A20 group*	67 (32)	149 (52)	87 (29)	154 (53)
*Lachnospiracea NK4A136 group*	9 (13)	175 (133)	184 (105)	228 (133)
*Lachnospiraceae UCG-001*	36 (16)	62 (51)	35 (12)	46 (11)
*Lachnospiraceae UCG-002*	-	-	-	5 (4)
*Lachnospiraceae UCG-007*	-	-	-	9 (13)
*Lachnospiraceae UCG-010*	106 (29)	58 (18)	101 (29)	154 (68)
*Lysinibacillus*	2 (4)	-	3 (7)	11 (1)
*Marvinbryantia*	16 (10)	157 (54)	27 (11)	158 (44)
*Mitsuokella*	-	10 (10)	-	-
*Mogibacterium*	9 (6)	3 (7)	12 (8)	4 (8)
*Moryella*	4 (4)	7 (4)	7 (5)	10 (5)
*Negativibacillus*	24 (13)	51 (12)	30 (10)	66 (15)
*Oscillibacter*	112 (26)	148 (25)	86 (15)	110 (22)
*Oscillospira*	66 (28)	1 (2)	14 (5)	1 (2)
*Paeniclostridium*	1,543 (261)	433 (191)	705 (472)	370 (174)
*Papillibacter*	11 (11)	4 (0)	14 (5)	3 (0)
*Phascolarctobacterium*	114 (41)	155 (31)	107 (18)	136 (42)
*Pseudobutyrivibrio*	9 (20)	-	-	-
*Pygmaiobacter*	29 (17)	69 (39)	10 (6)	56 (28)
*Romboutsia*	1,119 (206)	514 (370)	902 (462)	952 (423)
*Roseburia*	17 (10)	202 (257)	10 (8)	71 (91)
*Ruminiclostridium*	72 (29)	15 (10)	75 (28)	7 (2)
*Ruminococcaceae Eubacterium coprostanoligenes group*	795 (141)	535 (250)	880 (109)	928 (214)
*Ruminococcace GCA-90066225*	8 (4)	17 (10)	10 (6)	13 (6)
*Ruminococcace NK4A214 group*	217 (49)	123 (38)	275 (33)	187 (33)
*Ruminococcaceae UCG-002*	91 (25)	18 (13)	104 (29)	34 (20)
*Ruminococcaceae UCG-004*	58 (15)	45 (12)	59 (15)	60 (20)
*Ruminococcaceae UCG-005*	2,144 (288)	2,913 (396)	2,214 (395)	3,114 (387)
*Ruminococcaceae UCG-009*	209 (39)	84 (38)	238 (55)	122 (22)
*Ruminococcaceae UCG-010*	1,881 (181)	978 (439)	1,736 (192)	1,044 (525)
*Ruminococcaceae UCG-011*	26 (17)	-	110 (12)	-
*Ruminococcaceae UCG-013*	1,156 (150)	543 (274)	822 (83)	941 (218)
*Ruminococcaceae UCG-014*	302 (83)	343 (347)	360 (44)	363 (76)
*Ruminococcu gauvreauii group*	2 (5)	30 (24)	7 (9)	77 (27)
*Saccharofermentans*	10 (10)	3 (2)	36 (7)	11 (6)
*Sharpea*	-	16 (17)	-	18 (9)
*Solobacterium*	-	-	11 (8)	-
*Streptococcus*	1 (3)	2 (2)	4 (4)	1 (1)
*Syntrophococcus*	2 (4)	4 (10)	11 (12)	288 (229)
*Terrisporobacter*	39 (17)	18 (0)	18 (7)	-
*Turicibacter*	141 (19)	254 (273)	166 (70)	117 (100)
*Tyzzerella 4*	29 (17)	107 (87)	67 (21)	116 (32)
*XBB1006*	-	-	7 (8)	-
Uncultured Christensenellaceae	-	-	8 (6)	-
Uncultured Clostridiales vadinBB60 group	99 (26)	188 (202)	69 (37)	179 (108)
Uncultured Erysipelotrichaceae	12 (9)	25 (33)	-	16 (11)
Uncultured Lachnospiraceae	42 (18)	92 (47)	62 (12)	42 (6)
Uncultured Peptococcaceae	150 (20)	64 (13)	150 (55)	48 (16)
Uncultured Ruminococcaceae	239 (35)	92 (23)	252 (48)	115 (40)
Uncultured Veillonellaceae	-	-	-	14 (7)
Unclassified Firmicutes	1,019 (296)	2,092 (722)	1,128 (302)	1,882 (953)
*Patescibacteria*	62 (7)	37 (13)	83 (23)	113 (40)
*Candidatus Saccharimonas*	62 (7)	37 (13)	83 (23)	113 (40)
*Proteobacteria*	351 (108)	760 (456)	566 (197)	216 (144)
*Escherichia-Shigella*	-	39 (24)	-	6 (9)
*Kingella*	3 (5)	6 (13)	-	-
*Mailhella*	178 (60)	19 (9)	279 (47)	20 (13)
*Parasutterella*	47 (16)	52 (24)	23 (9)	28 (11)
*Ruminobacter*	3 (4)	54 (27)	25 (37)	16 (28)
*Succinivibrio*	9 (21)	512 (446)	1 (1)	117 (93)
*Succinivibrionaceae UCG-001*	-	5 (4)	-	-
*Sutterella*	-	9 (4)	-	3 (8)
Uncultured Paracaedibacteraceae	6 (2)	-	-	-
Uncultured Rhodospirillales	63 (33)	-	168 (133)	16 (24)
Uncultured Rickettsiales	6 (4)	7 (2)	-	-
Unclassified Proteobacteria	36 (24)	-	70 (80)	10 (14)
*Spirochaetes*	197 (44)	133 (89)	143 (35)	263 (129)
*Sediminisprirochaeta*	10 (8)	-	-	-
*Spirochaetaceae GWE-31-10*	20 (13)	9 (15)	14 (6)	37(9)
*Treponema*	167 (32)	124 (75)	129 (29)	226 (126)
*Tenericutes*	128 (45)	139 (19)	88 (37)	84 (26)
*Anaeroplasma*	-	40 (15)	-	9 (10)
Uncultured Tenericutes	113 (53)	82 (70)	70 (67)	58 (39)
Unclassified Tenericutes	15 (10)	17 (20)	18 (24)	17 (17)
*Verrucomicrobia*	481 (96)	82 (113)	850 (195)	379 (122)
*Akkermansia*	481 (96)	82 (113)	850 (195)	379 (122)

**Fig. 1:**
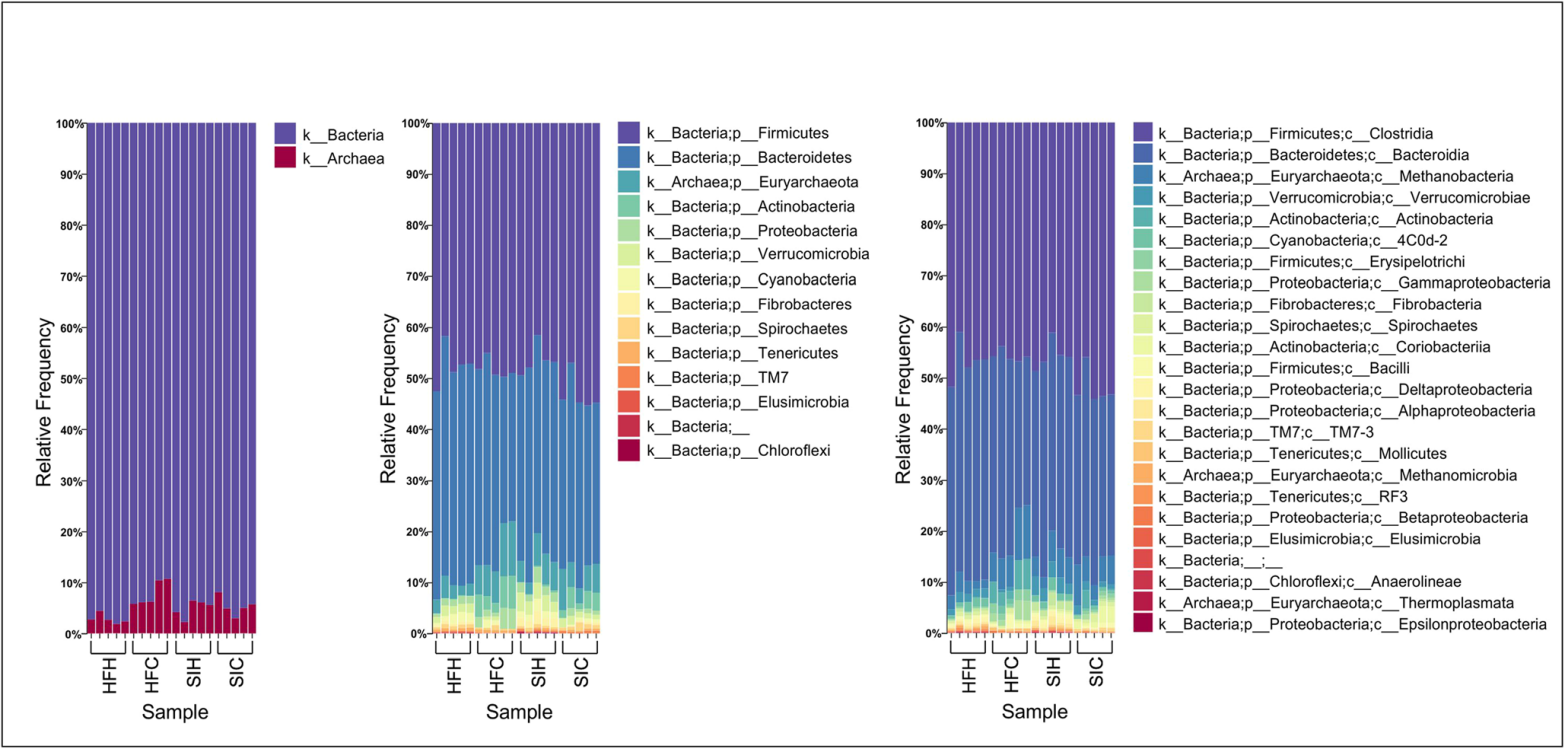
Next-generation sequencing (NGS) relative abundance at the kingdom, phylum, and class levels. The figure represents 5 experimental replicates for each experimental group (*x*-axis). Annotation was done using SILVA database: kingdoms are shown on the left side of the figure, phyla on the central part, and classes on the right side

Archaea were represented by *Euryarchaeota* phylum which includes 5 genera equally distributed among the 2 experimental groups (Table 2). Within this phylum, we found several microorganisms that colonize the rumen and are involved in methane production (Holmes and Smith 2016). It is worth noting that the difference regarding *Methanosphaera* genus was larger for HFC (141 sequences) and SIC (157 sequences) than HFH (22 sequences) and SIH (74 sequences), likely due to the physiological status and diet composition (Hook et al. 2010). As expected, Bacteria was the largest domain in all experimental groups, representing about two-thirds of the total microbiome (Fig. 1).

### Abundance of bacterial and archeal communities differs between heifers and lactating cows

As expected, within bacteria domain, *Firmicutes* represented the most abundant phylum (Table 2 and Fig. 1). In HFH, *Paeniclostridium* and *Romboutsia* were the largest genera, covering 1,543 and 1,119 sequences, respectively, followed by *Clostridium*, *Eubacterium*, and *Turicibacter*. The same order of abundance was maintained in SIH and SIC; however, in HFC, *Anaerovibrio* (97 sequences), *Blautia* (137 sequences), *Marvinbryantia* (157 sequences), *Oscillibacter* (148 sequences), *Roseburia* (202 sequences), and *Lachnospiraceae AC2044group* (274 sequences) showed high abundance. In SIH, the *Candidatus* class showed good number of sequences (138 sequences), while in SIC *Syntrophococcus* sequences were greater than the other groups. Moreover, *Fourneriella* genus was identified only in HFC (13 sequences) and SIC (12 sequences), being probably related to a diet rich in corn. *Intestinimonas* was represented only in HFH (3 sequences) and SIH (6 sequences).

*Bacteroidetes* represented the second most abundant phylum with 12,529 sequences in HFH; 10,308 in HFC; 9,997 in SIH; and 10,019 in SIC (Table 2). *Prevotellaceae* was the largest family and in all groups comprised more than 3,000 sequences, with a particular relative abundance of some genera in heifers, as *Prevotellaceae UCG-004*, or in cows, as *Prevotellaceae UCG-003*. *Rikenellaceae RC9 gut group* was the second largest genus and comprised 2,380 sequences in HFH; 2,015 in HFC; 2,238 in SIH; and 1,918 in SIC. The third largest genus identified was *Bacteroides* with high differences between heifers and cows; indeed, in HFH (1,715 sequences) and SIH (1,759 sequences), the abundance was lower compared with HFC (2,807 sequences) and SIC (2,350 sequences). *Alistipes* was another important genus (Table 2) that showed great variability among the experimental groups: 1,489 sequences in HFH; 866 in HFC; 1,108 in SIH; and 709 in SIC, and it is typical component of gut microbiome (Xin et al. 2019).

The third largest phylum was *Actinobacteria*; these Gram-positive bacteria have been studied mainly as a minor component of rumen microflora, representing about 3% of entire community, and this aspect reflect our results (Sulak et al. 2012). This group is involved in the amylase, caseins, gelatinase, lipase, chitins, and cellulose enzyme production (Borsanelli et al. 2018).

### Minor phyla

The incidence of *Proteobacteria* phylum was variable among the 2 experimental groups. In particular, 251 sequences were detected in HFH, 760 in HFC, 566 in SIH, and 216 in SIC (Table 2). *Succinivibrio* was the largest genus of *Proteobacteria* especially in HFC (512 sequences); the high presence of genus *Succinivibrio* in cows is probably related to an abundant corn-based diet as described by Kim et al. (2014). *Mailhella* genus was the largest phylotype in heifers (178 sequences in HFH and 279 in SIH); however, their role is almost unknown in cattle and probably their different abundance could be related to animal diet. It is worth noting that the genera of *Proteobacteria* phylum reported in our study were significantly different from those reported in the study of Kim and Wells (2016). *Chloroflexi*, *Cyanobacteri*a, *Elusimicrobia*, *Epsilonbacteraeota*, *Fibrobacteres*, and *Verrucomicrobia* comprised the classes that showed differences in number of sequences among the experimental groups (Table 2). Indeed, their abundance was higher in heifers than cows. *Chloroflexi* phylum was observed only in HFH (3 sequences) and SIH (34 sequences) due to contamination, since this phylum includes environmental photosynthetic bacteria (Borsanelli et al. 2018). *Elusimicrobia* and *Epsilonbacteraeota* are 2 phyla not commonly present in cattle feces (Kim et al. 2017), while Cyanobacteria have been reported in other species, but their role in cattle remains still unknown (Shepherd et al. 2012). *Fibrobacteres* phylum belongs to the group of bacteria that colonize the rumen; their function in cattle is related to fiber digestion, and thus, their presence is associated to a diet rich in forage (AlZahal et al. 2017). V*errucomicrobia* phylum was the most variable among the experimental groups. Indeed, we found 481 sequences in HFH, 82 in HFC, 850 in SIH, and 379 in SIC (Table 2). These bacteria are usually not affected by the different diets or sample fractions and their role is still unknown (Deusch et al. 2017). *Patescibacteria* and *Spirochaetes* are commonly present in feces of cattle (Nyonyo et al. 2014); indeed, their level has been considered constant among the experimental groups (Table 2). *Patescibacteria* function is still unknown, while *Spirochaetes* phylum is associated with the cellulolytic activity (Nyonyo et al. 2014).

### Alpha-diversity

**Fig. 2:**
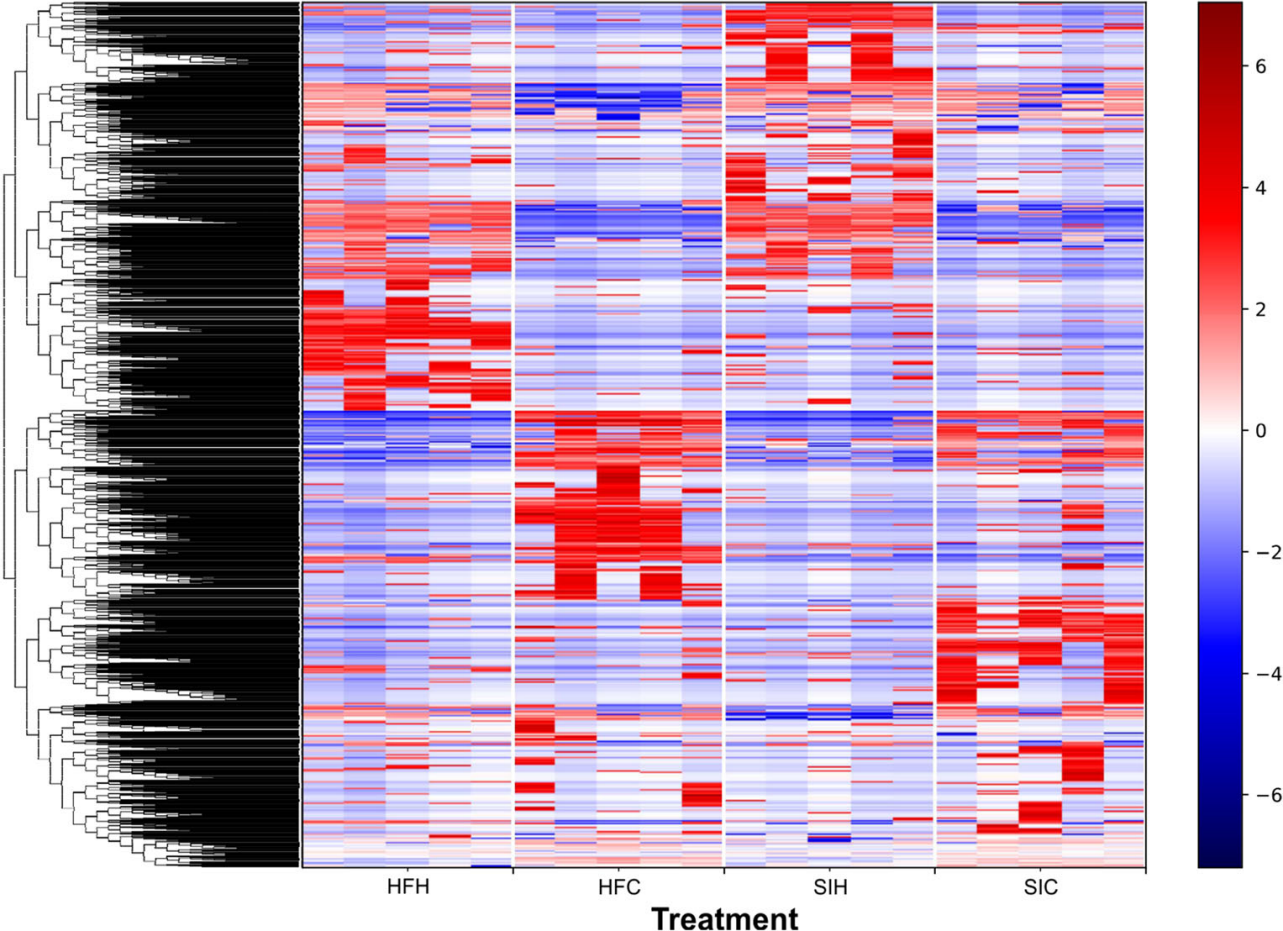
Hierarchical clustering of core OTUs in the 4 experimental groups, divided by quadrants. All core OTUs were clustered among the animals of the experimental groups. The blue background represents 0 counts, whereas red color indicates higher counts for that particular taxonomic unit in a specific sample

Results of α-diversity test are shown in Table 3. The number of OTUs for all experimental groups ranged from 676 to 798; in particular, the average number of identified OTUs ± SD in HFH, HFC, SIH, and SIC was 798 ± 144, 676 ± 75, 733 ± 88, and 716 ± 75, respectively. The number of observed OTUs was not statistically different among samples of the same breed, whereas significant differences were observed for the other indexes. Pielou’s Evenness Index showed differences (*p* < 0.05) between cows and heifers of the same breed: HFH (0.915 ± 0.003) vs HFC (0.883 ± 0.016), and SIH (0.918 ± 0.008) vs SIC (0.899 ± 0.007), related to a different abundance of species, due to the diet composition and animal category. Through Shannon Index the observed number of species was different only between HFH (32.970 ± 2.997) and HFC (28.557 ± 2.228) (*p* < 0.05). Faith’s Phylogenetic Diversity Index underlined that just the comparison between HFH (8.799 ± 0.241) and HFC (8.299 ± 0.234) was statistically significant (*p* < 0.05), proving that within Holstein-Friesian breed, the microbial species seem to have different phylogenetic taxon. Finally, the OTU table was normalized according to the OTU abundance across samples, and the results were clustered as the heatmap to provide a better pattern across the experimental groups (Fig. 2).

**Table 3:** Summary of 16S rRNA OTU data and α-diversity values (Evenness Index, Shannon Index, and Faith’s Phylogenetic Diversity Index) in association with statistical test

	Observed OTUs			Evenness Index			Shannon Index			Faith PD Index		
	Number	SD		Pielou_e	SD		Value	SD		Value	SD	
HFC	676	75		0.883	0.016		28.557	2.228		8.299	0.234	
HFH	798	144		0.915	0.003		32.970	2.997		8.799	0.241	
SIC	716	85		0.899	0.007		31.547	1.685		8.521	0.190	
SIH	733	88		0.918	0.008		31.916	1.421		8.725	0.180	
Kruskal-Wallis (pairwise)												
	H	*p* value	*q* value	H	*p* value	*q* value	H	*p* value	*q* value	H	*p* value	*q* value
HFC vs HFH	2.45	0.11	0.69	6.81	0.009	0.013	4.18	0.028	0.084	4.81	0.028	0.084
SIC vs SIH	0.99	0.75	0.75	6.81	0.009	0.013	2.45	0.117	0.140	0.098	0.754	0.754

Each index is reported as mean ± standard deviation of the calculated value for each animal within the experimental group. The statistical comparison was considered only between the experimental groups of the same breed through Kruskal-Wallis test

*HFC*, Holstein-Friesian cows; *HFH*, Holstein-Friesian heifers; *SIC*, Simmental cows; *SIH*, Simmental heifers

### Beta-diversity

Results of β-diversity were computed on the rarefied OTU table using different metrics (Table 4). The Bray-Curtis Metric showed high dissimilarity across experimental groups of the same breed, where the index was weakly high in the comparison HFH vs HFC (0.74), and SIH vs SIC (0.64). The PERMANOVA test underlined that the difference was statistically different between the samples of the same breed (HFH vs HFC and SIH vs SIC; *p* < 0.05). The Jaccard Metric provides the difference in the microbial composition among the experimental groups, HFH vs HFC (0.80) and SIH vs SIC (0.78), underlying a significant difference in fecal microbial composition (*p* < 0.05). The UniFrac Metric was considered unweighted and weighted (Table 4). The UniFrac Metric was low in the comparisons HFH vs HFC (unweighted 0.51 and weighted 0.23) and SIH vs SIC (unweighted 0.48 and weighted 0.21). Such results were supported by the statistical significance of the PERMANOVA test (*p* < 0.05) in inter-breed comparisons.

**Table 4:** β-Diversity values through Bray-Curtis, Jaccard, and unweighted and weighted UniFrac Metrics. *P* value for each comparison was obtained from PERMANOVA and considered significant at *p* < 0.05

	Value	Pairwise PERMANOVA result	Pseudo *F*	*p* value	*q* value
Bray-Curtis					
HFH-HFC	*0.74*	HFC vs HFH	10.9	0.009	0.013
SIH-SIC	*0.64*	SIC vs SIH	7.2	0.009	0.013
Jaccard					
HFH-HFC	*0.80*	HFC vs HFH	7.46	0.011	0.013
SIH-SIC	*0.78*	SIC vs SIH	5.75	0.008	0.012
Unweighted UniFrac					
HFH-HFC	*0.51*	HFC vs HFH	10.93	0.009	0.016
SIH-SIC	*0.48*	SIC vs SIH	8.28	0.012	0.016
Weighted UniFrac					
HFH-HFC	*0.23*	HFC vs HFH	17.27	0.012	0.014
SIH-SIC	*0.21*	SIC vs SIH	7.21	0.004	0.014

*HFC*, Holstein-Friesian cows; *HFH*, Holstein-Friesian heifers; *SIC*, Simmental cows; *SIH*, Simmental heifers. P-value for each comparison was considered significant at *p < 0.05*.

**Fig. 3:**
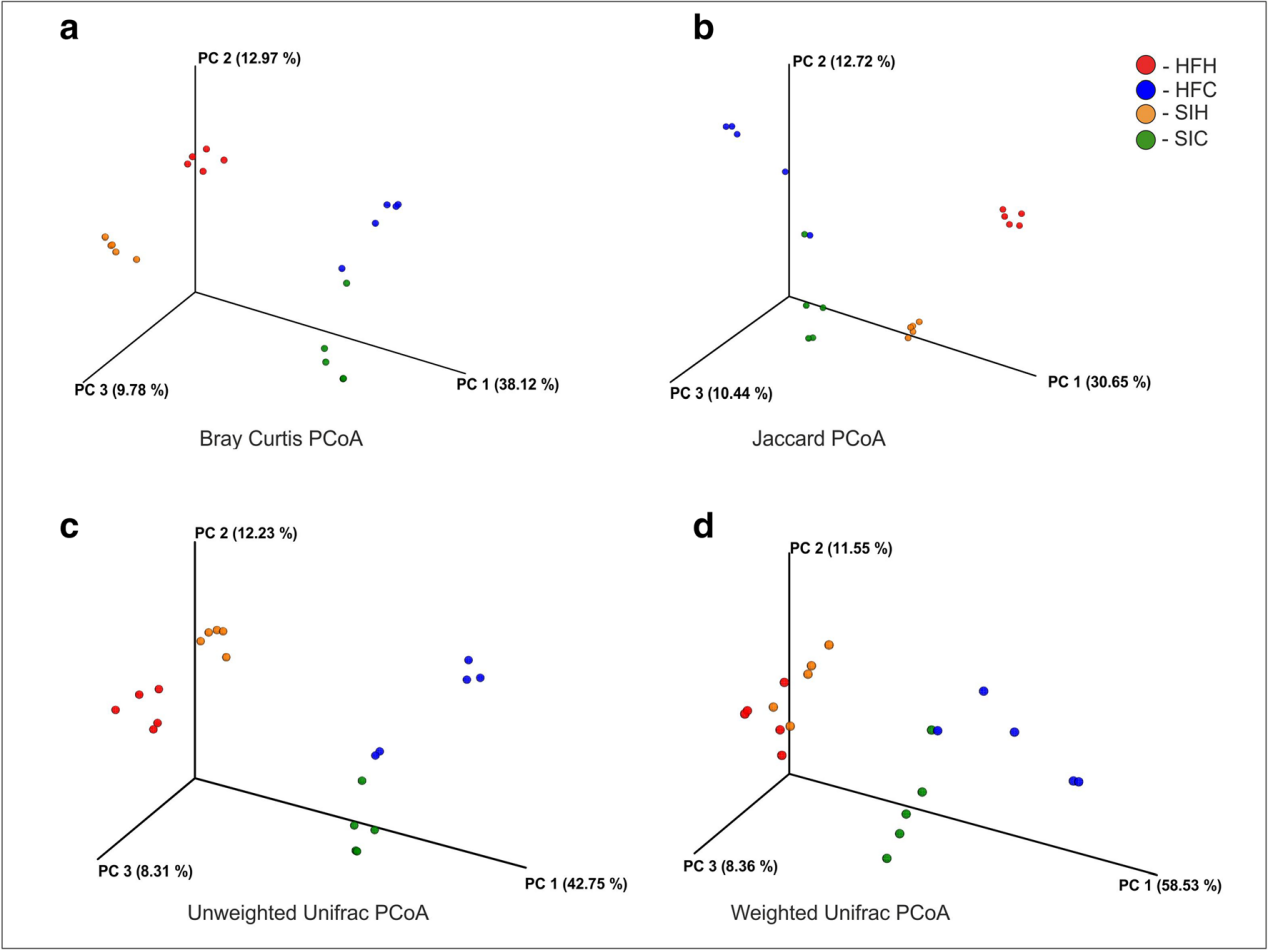
Principal coordinate analysis (PCoA) of 16S metagenomics data of microbial population in feces of Holstein-Friesian and Simmental heifers and cows. Comparison between community diversity based on different metric distances of microbial communities in cattle feces: **a** Bray-Curtis; **b** Jaccard; **c** unweighted UniFrac; **d** weighted UniFrac. Experimental groups are HFH (red), HFC (blue), SIH (orange), and SIC (green), each including 5 animals

PCoA of the Bray-Curtis, Jaccard, and unweighted UniFrac Metrics revealed that microbiota in fecal samples were clustered in 4 different groups identified as our 2 experimental groups (Bray-Curtis—PC1: 38.12%, PC2: 12.97%, PC3: 9.78%; Jaccard—PC1: 30.65%, PC2: 12.72%, PC3: 10.44%; unweighted UniFrac—PC1: 42.75%, PC2: 12.23%, PC3: 8.31%); the repeatability of the results of the 5 animals within the same group was very high, particularly among the HFH and SIH groups (Fig. 3). PCoA of the weighted UniFrac distance revealed that microbiota of the animals of the same group had high variability (weighted UniFrac—PC1: 58.53%, PC2: 11.55%, PC3: 8.36%) (Fig. 3).

### Abundance of species in comparative context: Holstein-Friesian heifers vs Holstein-Friesian cows and Simmental heifers vs Simmental cows

**Fig. 4:**
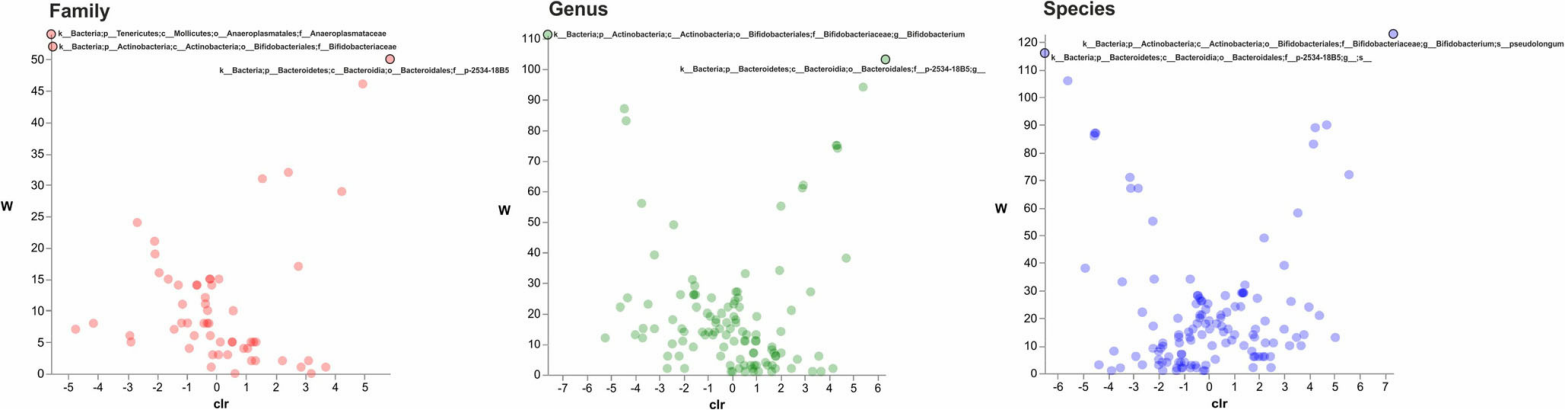
Volcano plot representation of ANCOM analyses. The horizontal axis is the centered log ratio transformation (clr) representative of the difference in abundance of a given taxonomical unit between Holstein-Friesian cows and Holstein-Friesian heifers at the family (red dots), genus (green dots), and species (blue dots) level. The W statistic indicates the value of the statistical test corresponding to the number of times the null hypothesis was rejected for each taxonomical class. Taxonomical classes with relative abundance significantly different (Holm-Bonferroni corrected *p* value < 0.05) are evidenced at the top of volcano plot and their taxonomy is reported. A darker shade of color indicates an overlap of OTUs

The analysis of composition of microbiomes (ANCOM) (Fig. 4) and volcano plot were performed in order to identify the relative abundance of species that was significantly different in the comparisons HFH vs HFC and SIH vs SIC (Kuczynski et al. 2012).

The comparison between HFH and HFC associated with the ANCOM statistical analysis detected significant differences for 3 bacterial families according to Holm-Bonferroni post hoc test (*p* < 0.05); *Anaeroplasmataceae* (W 54) and *Bifidobacteriaceae* (W 52) were the most important families in HFC, and p-2534-18B5 (W 50) in HFH (Fig. 4). The statistical analysis of genera abundance in HFH vs HFC showed significant differences (*p* < 0.05) for *Bifidobacterium* (*Bifidobacteriaceae*) (W 103) and for one unclassified genus (p-2534-18B5). Results of the lowest taxonomic level revealed significant differences of species (*p* < 0.05) between HFH and HFC, suggesting a greater presence of *Bifidobacterium pseudolongum* (W 123) in HFC and p-25434-18B unclassified bacterium (W 116) in HFH.

**Fig. 5:**
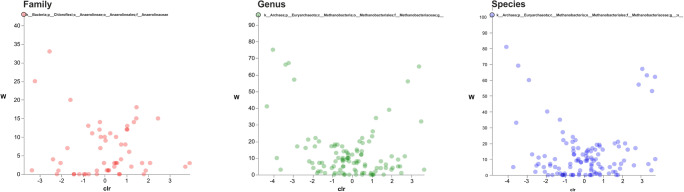
Volcano plot representation of ANCOM analyses. The horizontal axis is the centered log ratio transformation (clr) representative of the difference in abundance of a given taxonomical unit between Simmental cows and Simmental heifers at the family (red dots), genus (green dots), and species (blue dots) level. The W statistic indicates the value of the statistical test corresponding to the number of times the null hypothesis was rejected for each taxonomical class. The family, genus, and species with relative abundance significantly different (Holm-Bonferroni corrected *p* value < 0.05) are evidenced at the top of volcano plot and their taxonomy are reported. A darker shade of color indicates an overlap of OTUs

In Simmental breed, *Anaerolineales* family (W 43) and 1 unclassified *Methanobacteriaceae* genus (W 96) and species (W 101) showed significant differences (*p* < 0.05) when comparing heifers and cows (Fig. 5). *Anaerolinaceae* family was more abundant in histidine-fed heifers (Klevenhusen et al. 2017). Indeed, SIH showed more abundant archaeal *Methanocorpusculaceae* and bacterial *Elusimicrobiaceae* family. As reported for Holstein-Friesian breed, *Methanocorpuscolaceae* role in rumen has not been studied yet and the presence in feces could be related to a shift outside from the rumen.

## Discussion

In the present study, we assessed the diversity of microbiome in cow and heifer feces within Holstein-Friesian and Simmental cattle breeds. The 16S rRNA sequencing approach was used to perform analysis of bacterial communities. The members of *Firmicutes*, *Bacteroidetes*, *Proteobacteria*, and *Actinobacteria* phyla were the most abundant and showed great variability among experimental groups (Kim et al. 2014). The presence of genus *Fecalibacterium* in the HFC and SIC might be related to a high corn percentage in the diet fed to cows compared with the diet fed to heifers. This hypothesis is corroborated by the greater abundance of *Blautia* and *Roseburia* which seem to be correlated with a corn-rich diet and are involved in the production of butyrate; the latter plays a role in the energy source for the mucosa (Kim et al. 2014), similar to *Fecalibacterium* which promotes the main energy sources for the gut epithelial cells (Pryde et al. 2002). The most representative genera, in all experimental groups, were correlated to digest functions, such as *Clostridium*, *Eubacterium*, *Paeniclostridium*, *Romboutsia*, and *Turicibacter* as related to diet composition and probably to physiological traits (Fan et al. 2017; Gerritsen et al. 2017; Xin et al. 2019).

Results of α-diversity analyses showed a significant difference between HFH and HFC, which is probably related to animal categories and diet composition. Finally, the OTU table was normalized according to the OTU abundance across samples, and the results were clustered as the heatmap to provide a better pattern across the experimental groups (Fig. 2). There was high difference of the clustered OTUs across the experimental groups; nevertheless, we cannot compare the breeds due to different farming conditions (diet and management conditions).

The PCoA showed the presence of two distinct groups (Fig. 3), which represent heifers and cows; indeed, the microbiome seems to be notably different between these two animal categories. We can indirectly justify the microbiome difference between heifers and cows, since diet (Callaway et al. 2010; Fan et al. 2017; Xin et al. 2019) and lactation (AlZahal et al. 2017) are important factors for the determination of bacteriological composition of fecal community. The variation of data could be explained as there is evidence in scientific literature indicating that the composition of fecal microbiome is completely different from the rumen and the differences between the taxonomical groups were less in the fecal samples compared with rumen (Azad et al. 2019). The differences between HFH and HFC and between SIH and SIC are strongly marked, especially with regard to some microbial populations, such as *Tenericutes*, *Actinobacteria*, and *Bacteroidetes* between HFC and HFH, and *Chloroflexi* and *Euryarchaeota* between SIC and SIH. This variability can be explained by diet composition in relation to the physiological status of the animals. However, the statistical analysis identified families, genus, and species present just in a specific category and breed. The variability of feces microbiome between the animals of the same experimental group was low, denoting a good reproducibility within samples. Interestingly the taxonomic class seems to cluster between heifers and cows of the 2 different breeds. Even if we could not perform a direct comparison between breeds, we can note that the trend in cows and heifers is conserved. Therefore, we may hypothesize that this trend is fixed, even when different interactions among breeds and diets are considered. Recently, a specific study reported a genetic link between breed and rumen microbiome community (Sandri et al. 2018); thus, as future perspective, we will investigate the microbiome composition of feces samples from animals of different breeds under the same farming and diet conditions. Finally, microbiome composition is crucial to evaluate feed efficiency, animal welfare, and methane emissions from cattle (Oikonomou et al. 2013; Holmes and Smith 2016; Sandri et al. 2018). Indeed, several alterations of the microbiome can compromise the physiological functions of the animals, altering their production capacity and health. Thus, our study lays the groundwork for future investigations aimed at developing minimally invasive and routine screening system to collect information on microbiome composition at population level. This would open new opportunities to select for more efficient and healthy animals.
